# A novel application of strain sonoelastography can detect changes in Achilles tendon elasticity during isometric contractions of increasing intensity

**DOI:** 10.1186/s13047-019-0342-1

**Published:** 2019-05-21

**Authors:** Alessandro Schneebeli, Filippo Del Grande, Deborah Falla, Corrado Cescon, Ron Clijsen, Marco Barbero

**Affiliations:** 1 Rehabilitation Research Laboratory, Department of Business Economics, Health and Social Care, University of Applied Sciences and Arts of Southern Switzerland, SUPSI, Stabile Piazzetta, Via Violino, 6928 Manno, Switzerland; 2 Centre of Precision Rehabilitation for Spinal Pain (CPR Spine), School of Sport, Exercise Rehabilitation Sciences, College of Life Environmental Sciences, University of Birmingham, Birmingham, UK; 3 Servizio di Radiologia, Ospedale Civico e Italiano, Ente Ospedaliero Cantonale (EOC), Lugano, Switzerland; 4 Department of Business Economics, Health and Social Care, University of Applied Sciences and Arts of Southern Switzerland, SUPSI, Landquart, Switzerland; 5 University College Physiotherapy, Thim van der Laan AG, Landquart, Switzerland; 6 Faculty of Physical Education and Physical Therapy, Vrije Universiteit Brussel, Brussels, Belgium

**Keywords:** Strain sonoelastography, Achilles tendon, Elasticity

## Abstract

**Background:**

Mechanical and morphological properties of the Achilles tendon are altered in disease and in response to changes in mechanical loading. In the last few years different ultrasound based technologies have been used to detect tendon mechanical properties changes mainly in resting condition. Therefore the aim of this study was to evaluate if strain sonoelastography can identify changes in Achilles tendon elasticity during isometric contractions of increasing intensity.

**Methods:**

This cross-sectional study enrolled 37 healthy volunteers (19 women) with mean (±SD) age of 27.1 (±7.0) years between January and June 2017. Strain sonoelastography images of the Achilles tendon were acquired during an isometric ramp force (0 kg, 0.5 kg, 1 kg, 2 kg, 5 kg and, 10 kg). An external reference material was used to provide a comparison between the examined tissue and a material of constant elasticity. Friedman test with post hoc pairwise comparison were used to determine the correlation between the difference contraction levels.

**Results:**

The median and interquartile range (IQR) values for the strain ratio were 1.61 (1.5–2.9) in a relaxed state and 1.30 (1.07–2.02), 1.00 (0.76–1.66), 0.81 (0.70–1.19), 0.47 (0.39–0.73) and 0.33 (0.28–0.40) for 0.5 kg, 1 kg, 2 kg, 5 kg and 10 kg, respectively revealing increased tendon hardness with increasing contraction intensities. Friedman test revealed significant differences (*p* < 0.05) in the strain ratio between all contractions except between 0.5 kg – 1 kg (*p* = 0.41); 1 kg – 2 kg (*p* = 0.12) and 5 kg – 10 kg (p = 0.12).

**Conclusion:**

Strain sonoelastography can detect changes in Achilles tendon elasticity between different contraction intensities. The results provide an original force-elasticity curve for the Achilles tendon in a healthy, asymptomatic population.

**Trial registration:**

The study was approved by the Ethics Committee of Canton Ticino.

## Background

The Achilles tendon is the strongest tendon in the body and constitutes a dynamic link between the triceps surae and the calcanous bone [1, 2]. A high mechanical load is applied to the Achilles-gastrocnemius complex and tendon stiffness can increase in response to chronic load [3]. Changes in glycosaminoglycan content, reduction of collagen cross-linking content and alignment of collagen fibers are factors that may alter tendon elasticity [3].

Overuse injuries of the Achilles tendon can occur with excessive loading during physical exercise [4]. Repetitive overload and subsequent microtrauma can lead to inflammation of the tendon sheath or degeneration of the tendon body [5]. The mechanical and morphological properties of the Achilles tendon are altered in disease and in response to changes in mechanical loading, including a reduction in tendon stiffness and a softening of the tendon measured with sonoelastography in individuals with chronic tendinopathy [6, 7].

A healthy Achilles tendon with intact mechanical and morphological properties is expected to become stiffer and harder with increased tensile load during isometric contraction. [8, 9] As shown by Magnusson et al. [10], both deformation and strain of a healthy Achilles tendon increase with an increase in tendon isometric force. Similarly, Lersch et al. [11] demonstrated increased strain of the Achilles tendon when the tendon was subjected to an increased load. Wren et al. [12] showed an increased Young’s modulus in a similar in vitro investigation using a servo-hydraulic materials testing machine, and although in vivo and in vitro results are not directly comparable, the Achilles tendon seems to behave similarly.

Shear wave sonoelastography is based on acoustic radiation force impulses through tissue to obtain the elastic modulus (Young modulus, kPa) and is typically employed to measure Achilles tendon elasticity. An ultrasound pulse produces a directional shear wave within the tissues, and the propagation velocity of this wave is calculated using an ultrafast ultrasound tracking technique [13]. However, given that the upper limit of 800 kPa of Young modulus is often reached in the Achilles tendon when the ankle is near 0° of plantar flexion, this measurement technique is not suitable for the evaluation of the tendon in a functional position (near 0° plantar flexion), during usual movement or with additional load [14]. Moreover, signal saturation occurs when the Achilles tendon approaches the 0° plantar flexion or a dorsiflexed position and leads to an underestimation of the shear wave speed [15].

Strain sonoelastography is another ultrasound-based technique that enables the elasticity of musculoskeletal structures to be evaluated [16–18]. Tissue compression with an ultrasound probe is applied to a surface producing a displacement within the tissue. The displacement, which is less pronounced in harder than in softer materials, is calculated by comparing B-mode image pairs before and after compression [19]. A novel method using strain sonoelastography and calculation of a strain ratio was recently developed and provides a semi-quantitative evaluation of the tendon based on the elastography color scale [8]. Combined with an external reference material, this approach can identify changes between a contracted and relaxed state [8], however the accuracy of this novel method to detect small changes in tendon mechanical properties due to load, needs further investigation. The use of an external reference material with known elasticity properties allows the computation of the strain ratio between the tendon color scale and the external material which is not affected by changes during contraction. This method has established reliability [8, 20].

The added value of this methods is the combination of a measure of elasticity, provided by the transverse compression force of the probe, and the progressive longitudinal mechanical load given by the contraction of the triceps surae muscle.

A more comprehensive analysis of the Achilles tendon with evaluation of the tendon under load is warranted as this approach could provide a clearer indication about the functional impairment of tendon structure. Therefore, together with clinical history and standard imaging this technology could ultimately lead to an enhancement of the diagnosis and management of tendinopathy.

Thus, the aim of this study was to evaluate if strain sonoelastography using the strain ratio, can accurately detect changes in Achilles tendon elasticity during isometric contractions of varying intensity in healthy volunteers.

We hypothesized that differences between the selected contraction levels could be identified by a change in strain ratio of the sonoelastography color scale.

## Methods

### Study population

Thirty-seven healthy participants (19 women) were prospectively enrolled in the study between January to June 2017. The mean (±SD) age, height and weight were 27.1 (±7.0) years, 172.2 (±9.8) cm and 67.5 (±13.2) kg, respectively.

Inclusion criteria for the selection of the sample were: no history of tendon and/or foot injury or surgery; any painful episodes in the lower limb in the last year. Exclusion criteria were: history of connective tissue, endocrine or metabolic diseases; systemic inflammatory disorder; spondyloarthropathy, rheumatoidarthritis, or hypercholesterolemia. Furthermore, those usually taking estrogen or steroid treatment were excluded.

The study was approved by the Ethics Committee of Canton Ticino, conducted according to the Declaration of Helsinki and written informed consent forms were signed by participants. The report of this study adheres to STROBE (Strengthening The Reporting of Observational Studies in Epidemiology) guidelines.

Mean and standard deviation values of relaxed and contracted strain ratios from a previous study [8] using the same experimental setup were used to calculate the minimal sample size necessary to ensure a statistical power of 90% at the 5% significance level for a two-tailed hypothesis test. The Cohen’s d effect size has been calculated using the pooled standard deviation of the previous study [8]. Effect size was very large (d = 1.69). The calculation, performed with STATA 14 (College Station, TX, USA), showed that the minimal sample size of *n* = 32 was required.

### Ultrasound and sonoelastography

A MyLab Class C ultrasound device (Esaote, Genoa, Italy) with a linear probe (3–13 MHz) and elastography software were used to perform the measurements. The Achilles tendon was visualized through a custom made external material (Zerdine® CIRS, Norfolk, VA, USA). The properties of the reference material were tested by the manufacturer, the elasticity was 102 kPa, speed of sound was 1580 m/s, and the attenuation coefficient was 0.5 dB/cm/MHz. The external reference material was utilized to guarantee a comparison between the Achilles tendon and a material with known elasticity (102 kPa). Furthermore, the reference material was used as stand-off pads to create a steady contact between the transducer and the skin and to enhance the stability of the sonographer’s hand [21].

Light and rhythmic perpendicular compression was applied by the operator with the ultrasound probe on the reference material. A quality indicator graph on a screen in front of the operator was controlled to regulate the amount of the compression across condition and participants. Compression–relaxation cycles were provided for 5 s (approximately 100 frames) for each acquisition, during this time the sonoelastogram remained as stable as possible.

### Dynamometry

**Fig. 1:**
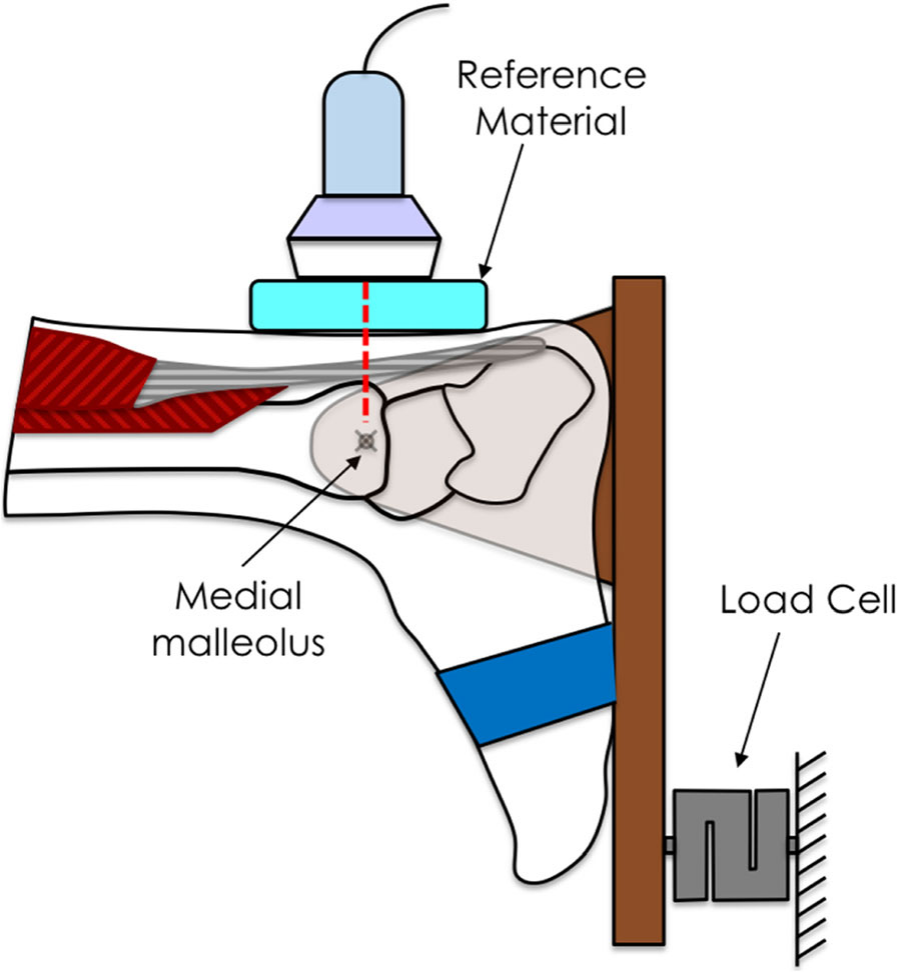
Schematic of the experimental setup. The foot was securely attached to a dynamometer with a wooden board at a fixed angle of 0° of dorsiflexion. The force measured by the load cell was proportional to the torque exerted at the ankle level. The ultrasound probe was placed in a longitudinal scan at the level of the medial malleolus

The torque of the ankle was assessed using a dynamometer connected to a force sensor that operated linearly in the range between 0 and 100 N (Mod. TF2/S; CCT Transducers, Turin, Italy). The force sensor was attached to a wooden board connected to a hinge that allowed natural dorsiflexion and plantar flexion of the ankle (Fig. 1). The board was fixed in order to permit isometric contractions only at a fixed angle of 0° dorsiflexion. The force measured by the force sensor was proportional to the torque exerted at the ankle level. Force signals were amplified using MISO-II (OT Bioelettronica, Turin, Italy; bandwidth 0–80 Hz) and presented to the participants as a real time visual feedback (60 Hz refresh rate) on a PC monitor positioned 50 cm in front of the subjects.

### Procedure

Participants were asked to lie in a prone position with the foot tightly secured within the dynamometer with the ankle in a neutral position (0° of plantarflexion). Prior to the recordings, the participants practiced the contraction with the instruction to “push on the board as if you were pushing onto the top of your toes”) and a contraction of 10 kg (maximum strength required) was performed to verify that the heel remained in contact with the dynamometer and to familiarize the participant with the visual feedback. Heel movement was prevented during the contraction by firmly securing the foot in the dynamometer with two belts over foot and ankle.

Both the left and right Achilles tendon of each participant were evaluated during isometric plantar flexion under increasing contraction intensities (0 kg, 0.5 kg, 1 kg, 2 kg, 5 kg and, 10 kg) performed in a randomised order. Strain sonoelastography of the Achilles tendon, was performed in a longitudinal scan using the Elasto-Dual software (Fig. 3). The external reference material was included in the image and the measurement were performed at the level of the medial malleolus applying an established and reliable method [8].

To guarantee the evaluation of the same tendon part under the different conditions, a reference point on the medial malleolus was drawn and the ultrasound transducer was aligned with the point each time. All the ultrasound procedures were conducted by the same, well trained operator, with 8 years of ultrasound and elastography experience.

The colour scale of the elastograms ranged from red, showing soft tissue, to blue showing hard tissue, whereas green to yellow indicating medium tissue elasticity. The elastograms were automatically constructed maintaining the same ideal settings during the entire study, as recommended by Havre et al. [22] in a previous study.

### Image analysis

The analysis of the 5 s ultrasound clip was leaded using a customized MATLAB® software (MathWorks, Natick, MA, USA). Two different regions of interest (ROI) were drawn on the sonoelastographic images, including the reference material and the Achilles tendon (Fig. 3). The range between soft and hard (from red to blue) was divided into 256 levels (0–255) according to the depth of colour of the sonoelastographic image where blue corresponds to 0 and red to 255. The frames of the entire clip were averaged to extract a single image where the colour distribution was calculated. The median and interquartile range of colours was computed for each acquisition.

Strain ratios were calculated between the Achilles tendon median range of colours and the external reference material median range of colours, comparisons between the different contraction levels were computed for each tendon.

### Statistical analysis

Statistical Package for the Social Sciences (SPSS) version 22 (SPSS, Chicago, IL, USA) was used to perform statistical analysis. Descriptive statistics (mean ± SD) were used to describe the characteristics of the sample. Shapiro-Wilk test was used to evaluate if the data were normally distributed. Non-parametric, independent samples, Mann-Whitney test was then used to assess whether there were significant differences in Achilles tendon elasticity between men and women and between the right and left Achilles tendon.

Since the data were not normally distributed, Friedman test with post hoc pairwise comparison (Wilcoxon signed-rang test) were used to determine the correlation between elasticity measured at different contraction levels. Bonferroni correction was applied and statistical significance was set to α = 0.05.

Data are presented as median and interquartile range (IQR).

## Results

No significant difference in Achilles tendon strain ratios was observed between men median (IQR) 1.31 (0.96–1.81) and women 2.00 (1.19–2.81) (*p* = 0.6) or between the left 1.48 (1.13–2.06) and right Achilles tendon 1.71 (0.94–2.91) (*p* = 0.5), therefore the data were pooled and further analysed as an entire sample.

**Fig. 2:**
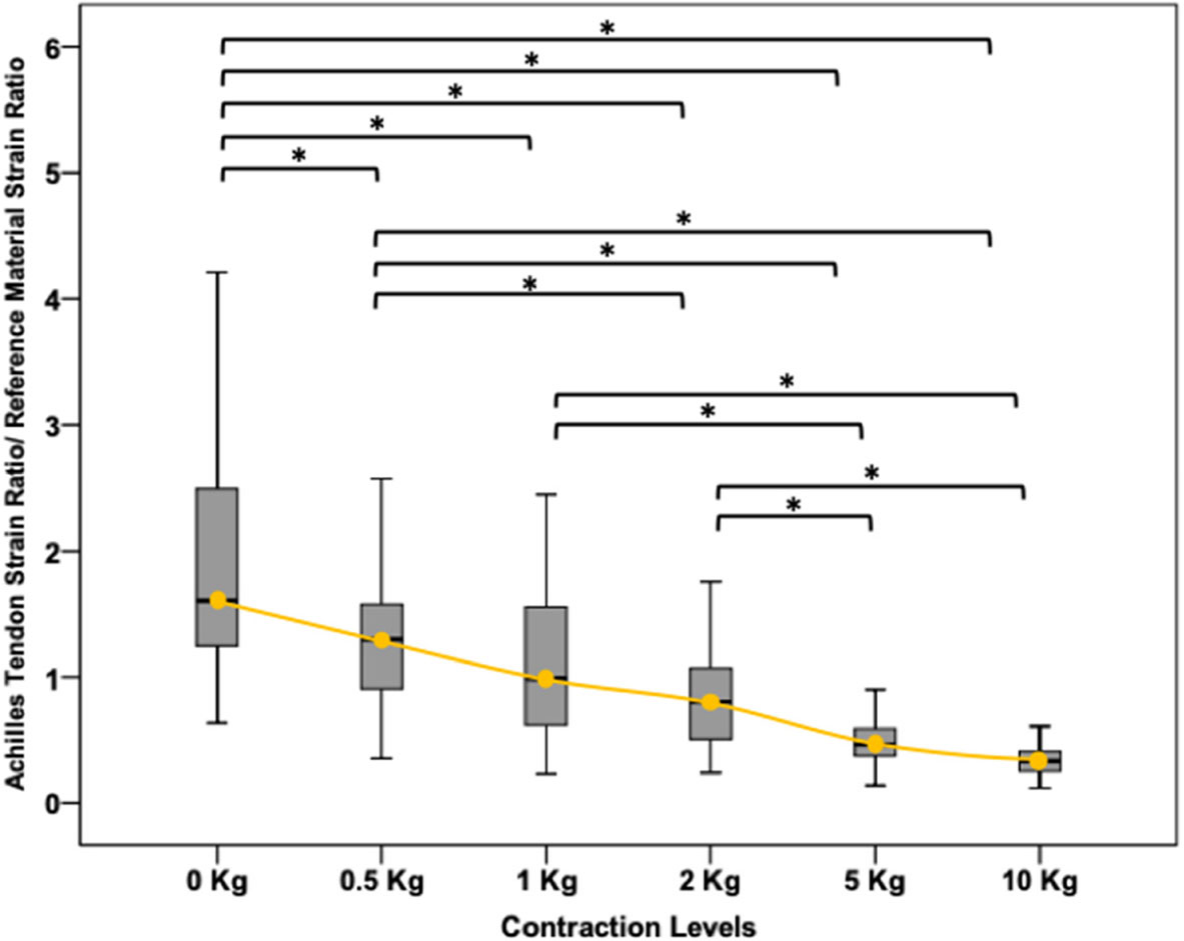
Force-elasticity curve. Box plot showing the median and interquartile range of values of the entire sample during the different contraction levels. The orange line represent the force-elasticity curve. * *p* < 0.01; statistical significant difference between the different contraction levels

The median (IQR) values for the strain ratio were 1.61 (1.5–2.9) in a relaxed state and 1.30 (1.07–2.02), 1.00 (0.76–1.66), 0.81 (0.70–1.19), 0.47 (0.39–0.73) and 0.33 (0.28–0.40) for 0.5 kg, 1 kg, 2 kg, 5 kg and 10 kg, respectively. Friedman test for related samples showed significant (*p* < 0.01) differences between all contraction levels except for 0.5 kg – 1 kg (*p* = 0.41); 1 kg – 2 kg (*p* = 0.12) and 5 kg – 10 kg (p = 0.12) (Fig. 2). Detailed pairwise comparison for all the contraction levels are presented in Table 1.

**Table 1:** Pairwise comparison for the different contraction levels. Statistical significant results (*p* < 0.01) are represented in bold

Contraction levels (Kg)	Pairwise comparison	Contraction levels (Kg)	Pairwise comparison	Contraction levels (Kg)	Pairwise comparison	Contraction levels (Kg)	Pairwise comparison	Contraction levels (Kg)	Pairwise comparison
0–0.5	***p*** **< 0.01**	0.5–1	*p* = 0.41	1–2	*p* = 0.12	2–5	***p*** **< 0.01**	5–10	*p* = 0.12
0–1	***p*** **< 0.01**	0.5–2	***p*** **< 0.01**	1–5	***p*** **< 0.01**	2–10	***p*** **< 0.01**	–	–
0–2	***p*** **< 0.01**	0.5–5	***p*** **< 0.01**	1–10	***p*** **< 0.01**	–	–	–	–
0–5	***p*** **< 0.01**	0.5–10	***p*** **< 0.01**	–	–	–	–	–	–
0–10	***p*** **< 0.01**	–	–	–	–	–	–	–	–

## Discussion

This is the first study to evaluate changes in Achilles tendon elasticity using strain sonoelastography during progressive increases in contraction force. The results provide an original force-elasticity curve for the Achilles tendon in a healthy, asymptomatic population (Fig. 2).

This study confirms that strain sonoelastography can detect changes in Achilles tendon elasticity between a relaxed state and all the examined contraction levels; the Achilles tendon progressively become harder across the different contraction levels, and this increment was seen as a reduction of the strain ratio as previously reported in other studies [8, 9]. A previous study [8] with a similar experimental setup showed large variability of the Achilles tendon strain ratio between healthy subjects when measured in the resting state. This variability, similarly to our study, is reduced when load is put on the tendon (see Fig. 2). The large variability seen between participants when the tendon is measured in the relaxed state is likely related to variation in physical activity levels, age and individual anatomical differences. Although tendon elasticity changed across contraction intensities, the contraction levels with similar intensity (0.5 kg – 1 kg (*p* = 0.41); 1 kg – 2 kg (*p* = 0.12) and 5 kg – 10 kg (p = 0.12)) revealed similar measures of elasticity thus likely the difference in load was insufficient to change tendon mechanical properties to a significant extent.

**Fig. 3:**
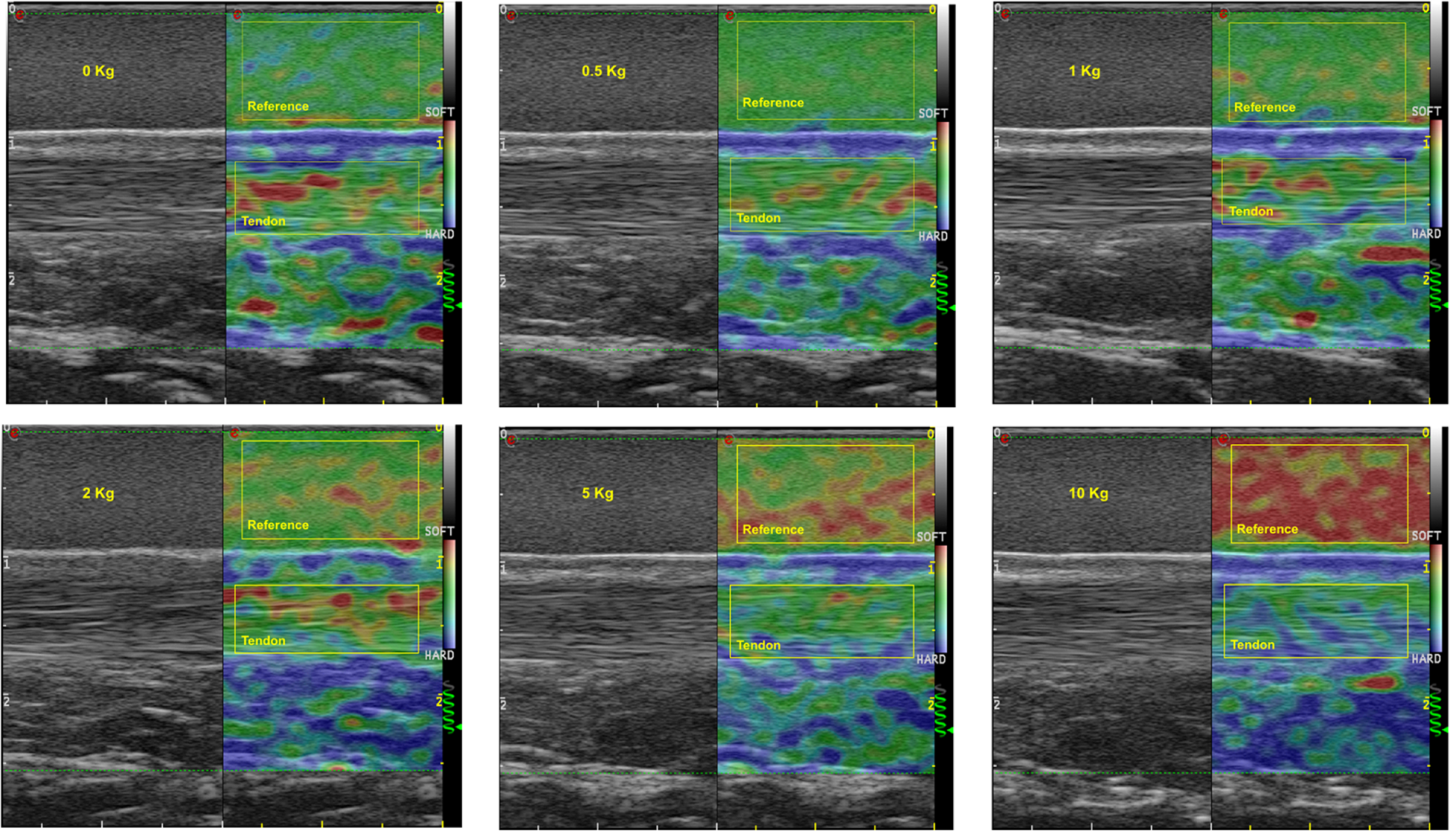
Achilles tendon sonoelastography during an isometric ramp contraction. Elasto-Dual images of the Achilles tendon during the isometric ramp contraction; the regions of interest (i.e., yellow boxes) define the two tissues examined for the calculation of the strain ratio. The six different boxes represent the different contraction levels

Previous studies [23–26] used the Kager Fat pad (KFP) as a medium to calculate the strain ratio in the evaluation of Achilles tendon elasticity. The results showed moderate to good reliability [24] and the authors suggested the use of strain elastography in the evaluation of the Achilles tendon following surgery [23]. However, caution should be taken when measuring strain ratio with a biological tissue such as KFP since other tissues could encounter changes in elasticity due to pathology, recovery or to different measurement conditions. The mechanical properties of the KFP changes following the hardening of the Achilles tendon and this change is not always linear, likely because of anatomical differences between subjects e.g. longer soleus muscle, more connective tissue, or due to the compression of the Achilles tendon on the underlying tissues. In the current study, we used an external material with a constant elasticity. Figure 3 shows an apparent softening of the reference material as the tendon became harder. The sonoelatography algorithm implemented in the ultrasound machine display the elasticity of the tendon with an “auto-scale” color range. The softest and the hardest structure visible in each of the ROI analyzed are displayed in red and blue respectively. For this reason, we can observe a progressive color change of the reference material (green at 0 kg, red at 10 kg) (see Fig. 3) because the tendon progressively becomes the harder structure within the ROI. However, strain ratios (i.e. ratios between the median colors) have been used as an index of elasticity which is not affected by the “auto-scale” color range.

The findings of the present study are supported by other investigations which measured strain of the Achilles tendon using a strain tracking procedure during maximal voluntary contractions [10, 27]. However, in these studies tendon displacement and tendon strain were measured using the aponeurosis-tendon complex while in the present study we have characterized changes in tendon elasticity with a direct measure of the free tendon. Other ultrasound-based techniques with direct measures of the tendon (i.e. shear wave elastography) have measured an increase of Young modulus (kPa) while the tendon is tensioned by changing the degree of ankle flexion [14, 15]. However in those studies it was suggested that the use of shear wave elastography should be limited to the relaxed tendon to avoid saturation of the signal.

Another promising 2D ultrasound technology, based on a speckle tracking algorithm [28] has been proposed. A non-uniform regional strain behavior of the different layers of the Achilles tendon has been observed. However given the different nature of strain sonoelastography measurement compared to the speckle tracking technique, the results cannot be directly compared.

Future studies which apply strain sonoelastography to evaluate the free tendon in different ankle positions or under different conditions (i.e. varying contraction intensity; functional tasks) are now warranted to better understand and characterize the behavior of the Achilles tendon. Moreover, the addition strain sonoelastography measures of the Achilles tendon during loaded contractions may increase diagnostic capability in Achilles tendinopathy and future research is needed to explore this.

Strain sonoelastography requires manual compression and therefore variation in the level of pressure of the transducer on the skin may affect the results. To reduce such variability, both very high and low pressure was avoided as proposed by Itoh et al. [29] and visual feedback of the applied pressure was provided so that it could be constantly controlled by the experienced operator.

Furthermore, strain ratio employed in this study offers a measure of elasticity provided by a transverse compression force of the ultrasound probe on the tendon structure. Further research is needed to establish the relationship between strain ratio and tendon longitudinal mechanical properties.

## Conclusions

Strain sonoelastography can detect changes in Achilles tendon elasticity between different contraction intensities. It was observed that tendon elasticity was not modified between some contraction intensities. The reduction of the strain ratio between the tendon and the reference material indicates a constant increase of tendon elasticity during contractions of increasing intensity and provides an original force-elasticity curve. Future studies, using strain sonoelastography with an external reference material, are now warranted to explore potential changes in Achilles tendon elasticity in those with tendinopathy.

## Abbreviations

KFP: Kager Fat Pad
ROI: Region of Interest
